# Understanding the Athena SWAN award scheme for gender equality as a complex social intervention in a complex system: analysis of Silver award action plans in a comparative European perspective

**DOI:** 10.1186/s12961-020-0527-x

**Published:** 2020-02-14

**Authors:** Evanthia Kalpazidou Schmidt, Pavel V. Ovseiko, Lorna R. Henderson, Vasiliki Kiparoglou

**Affiliations:** 1 Department of Political Science, Aarhus University, Aarhus, Denmark; 2 Radcliffe Department of Medicine, Medical Science Division, University of Oxford, Oxford, United Kingdom; 3 NIHR Oxford Biomedical Research Centre, Oxford, United Kingdom; 4 Nuffield Department of Primary Care Health Sciences, Oxford, United Kingdom

**Keywords:** Athena SWAN, gender equality award scheme, medical sciences, complexity approach, typology of interventions, European comparative, responsible research and innovation

## Abstract

**Background:**

Given the complex mix of structural, cultural and institutional factors that produce barriers for women in science, an equally complex intervention is required to understand and address them. The Athena SWAN Award Scheme for Gender Equality has become a widespread means to address barriers for women’s advancement and leadership in the United Kingdom, Ireland, Australia, the United States of America and Canada, while the European Commission is exploring the introduction of a similar award scheme across Europe.

**Methods:**

This study analyses the design and implementation of 16 departmental Athena SWAN Silver Action Plans in Medical Sciences at one of the world’s leading universities in Oxford, United Kingdom. Data pertaining to the design and implementation of gender equality interventions were extracted from the action plans, analysed thematically, coded using categories from the 2015 Athena SWAN Charter Awards Handbook and synthesised against a typology of gender equality interventions in the European Research Area. The results were further analysed against the complexity research literature framework, where research organisations are perceived as dynamic systems that adapt, interact and co-evolve with other systems.

**Results:**

Athena SWAN is a complex contextually embedded system of action planning within the context of universities. It depends on a multitude of contextual variables that relate in complex, non-linear ways and dynamically adapt to constantly moving targets and new emergent conditions. Athena SWAN Silver Action Plans conform to the key considerations of complexity – (1) multiple actions and areas of intervention with a focus on the complex system being embedded in local dynamics, (2) the non-linearity of interventions and the constantly emerging conditions, and (3) impact in terms of contribution to change, improved conditions to foster change and the increased probability that change can occur.

**Conclusions:**

To enact effective sustainable structural and cultural change for gender equality, it is necessary to acknowledge and operationalise complexity as a frame of reference. Athena SWAN is the single most comprehensive and systemic gender equality scheme in Europe. It can be further strengthened by promoting the integration of sex and gender analysis in research and education. Gender equality policies in the wider European Research Area can benefit from exploring Athena SWAN’s contextually embedded systemic approach to dynamic action planning and inclusive focus on all genders and categories of staff and students.

## Background

Given the complex mix of structural, cultural and institutional factors that produce barriers for women in science, an equally complex intervention is required to address them [1]. It has been argued that gender inequalities persist due to “*culture, processes and practices that constitute the structural systems of contemporary organizations and therefore are taken for granted and mostly left unchallenged*” [2–4]. Several studies also reveal how deep-rooted assumptions about academia being a meritocracy reproduce inequalities and call for scrutinising the existing practices, procedures and structures [5–8]. While the structure of academic organisations reproduces gender stereotypes, such as power relations, the distribution of women and men at top level positions, career prospects, etc. [9–11], policy-makers and academic leaders have, for a long time, failed to recognise and address the institutionalised structural and cultural barriers that hinder women’s advancement and leadership in academic and research organisations [5]. In the last decade, the focus on structural and cultural barriers has gained prominence and has been successively “*built into the funding schemes of different agencies supporting interventions to reduce gender inequality in science*” [12]. Most notably, the Athena SWAN Charter globally, and the gender equality policies in the European Research Area, provide support and incentives for academic and research organisations to address structural and cultural barriers to women’s advancement and leadership through action planning.

The Athena SWAN Charter was established in the United Kingdom in 2005 to encourage and recognise the commitment of higher education and research institutions to advancing the careers of women in science and, in 2015, it was expanded to arts, humanities, social sciences, business and law [13]. When institutions join the Athena SWAN Charter, they commit to systematically assessing and advancing gender equality through action planning and applying for awards recognising their success. Applications are peer reviewed by academics, subject experts, human resources, and equality and diversity practitioners from other member institutions who then make recommendations on the level of awards [13], wherein a Bronze award requires an assessment of gender equality and the related challenges as well as a 4-year action plan to address these challenges; a Silver award recognises the successful implementation of the proposed action plan and its measurable impact; and a Gold award recognises beacons of achievement in gender equality and champions in promoting good practice in the wider community.

Athena SWAN has become a common means to address barriers for women’s advancement and leadership in the United Kingdom, Ireland and Australia; the United States of America and Canada use modified approaches. Discussions are underway in India, Japan and New Zealand. In the United Kingdom, the National Institute for Health Research (NIHR) requires the Athena SWAN Silver award as a prerequisite for applying for competitive biomedical research centre funding [14]. In Ireland, by 2023, higher education and research institutions will be required to hold the Athena SWAN Silver award to be eligible for competitive government research funding [15]. In Australia, the Australian Academy of Science and the Australian Academy of Technology and Engineering run the Athena SWAN gender equality award scheme as part of the Science in Australia Gender Equity programme [16]. In the United States, the American Association for the Advancement of Science uses a modified Athena SWAN self-assessment and improvement framework as part of its STEM Equity Achievement (SEA) Change programme [17]. In Canada, the government has committed to implementing a ‘made-in-Canada’ Athena SWAN initiative [18].^1^ India and Japan have also expressed interest in trialling the Athena SWAN framework [19].

A number of empirical studies have examined the positive impact as well as the limitations and unintended consequences of the Athena SWAN Charter in United Kingdom higher education institutions [14, 20–28]. With regard to positive impact, participation in the Athena SWAN Charter is associated with increased awareness of gender inequity and broader diversity issues [14, 21, 22]; challenges to discrimination and bias [14, 21]; improvements in women’s visibility, self-confidence and leadership skills [21]; an enhanced work environment and institutional support for women’s careers [14, 24]; increased appreciation of a work–life balance and caring responsibilities [14, 21, 24]; new mentoring and professional development opportunities for all staff [14, 21]; and, overall, the creation of a more supportive and inclusive university culture [20]. The limitations and unintended consequences of participation in the Athena SWAN Charter include perceptions of an administrative burden on institutions [23]; that women are undertaking a disproportionate amount of Athena SWAN work [14, 23, 25, 28]; resentment about perceived positive discrimination by some men [14]; belief that achieving the award could become an end in itself [14]; limited ability to address longstanding tenure, power and pay imbalances in a short period of time [14, 22]; and “*competing inequalities*” [26], whereby gender takes prevalence over race and white middle class women become the main beneficiaries of the Athena SWAN Charter [27].

In the wider European Research Area, three strategic objectives for fostering gender equality in research and innovation are promoted to achieve an increase in the share of women active in research and in leadership positions, and the integration of the gender dimension in research and curricula [29]. The development and implementation of gender equality action plans is a key instrument for promoting structural and cultural change in the European Research Area. Although the European Commission (EC) adopted the structural change approach as late as 2011 [30], efforts have significantly intensified in recent years, particularly following the introduction of the Responsible Research and Innovation approach [31].^2^ A number of multinational European projects focus on the implementation of gender equality action plans tailored to research institutions in a number of European countries producing a substantial knowledge base and establishing a set of best practices. Yet, in some countries, the development of gender equality action plans still remains in an embryonic stage, and in some others, where implementation is underway, institutional change needs to be further promoted and evaluated [32, 33]. To further activate cultural and structural change in research organisations and universities across Europe, the European Commission explores scenarios for the introduction of a gender equity award scheme https://cordis.europa.eu/project/id/872113 similar to Athena SWAN [34].

In this paper, based on the most recent research [12, 35] and rich empirical data, we argue that, to activate effective gender equality structural and cultural change, it is necessary that interventions acknowledge and operationalise the notion of complexity as their frame of reference. In this paper, we first present our methods, followed by a focus on the design and implementation of 16 Athena SWAN Silver action plans in medical sciences at one of Europe’s leading universities – Oxford University – analysing gender equality interventions thematically and presenting the results in a comparative European perspective using the complexity approach. Finally, we discuss Athena SWAN as a complex social intervention, formulating practical implications for implementation and impact assessment of Athena SWAN and other complex gender equality interventions, and outlining strategic opportunities to strengthen gender equality policies in the European Research Area.

### Complexity approach

Under the complexity approach [36, 37], interventions are considered as part of the complex system within which they take place. A complex system is characterised by a multitude of components, which, through continuous interaction, create a system-level organisation with the whole greater than the sum of its components and by positive feedback processes, in which outcomes of the process are necessary for the process itself [38]. Typically, a complex system operates in a multi-layered context and dynamically adapts in response to changes in the environment. In complex systems, there is a multitude of contextual variables that interact in complex, nonlinear ways [36]. “*Complex systems are adaptive—they respond to changes. This central feature of complex systems is what makes them distinct from systems that are merely complicated*” [36]. Explaining the difference between a complicated and a complex system, the authors state that an intervention composed of multiple components may not necessarily be complex, it may solely be complicated, and provide an illustrative example – sending a rocket to the moon is complicated (because it pre-requires specific skills and several components) but can be separated into sets of actions, which are anticipated, stable and linear. In contrast, raising a child can be complex, due to the emergent and non-linear nature of the relationship between actions and outcomes because children and parents are active agents with unpredictable behaviour that cannot be isolated from the wider context, i.e. family and society.

Complexity in research organisations is a dynamic complex framework that adapts, interacts and co-evolves with other systems, rather than being a stable arrangement of different contextual features [35, 39, 40]. What embodies complexity in complex interventions are disproportionate interactions (e.g. a proportionally small intervention can make a vast difference at some point in time), recursive causality with strengthening loops, and emergent outcomes that need to be addressed instantaneously [37]. Greenhalgh and Papoutsi [40] conclude that, instead of a linear, cause-and-effect causality, the complexity paradigm is characterised by “*emergent causality: multiple interacting influences account for a particular outcome but none can be said to have a fixed ‘effect size’*” as well as a pragmatic adaptation to changing contexts and emerging circumstances. Another aspect of complexity that helps us understand and manage variations across local contexts is self-organisation [41]. Manifestation of self-organisation takes place through specific action patterns that depend on locally available resources and local contextual conditions that can promote or impede successful implementations [42]. Chandler et al. [43] claim that introducing change in settings where particular medical practices are involved will be met by self-organisation processes that will work against system change and towards status quo and stability [44]. Operationalising the notion of complexity as a frame of reference thus implies multiple areas of intervention with a focus on the local dynamics [35].

By embracing complexity as a frame of reference [45] for design, implementation and impact assessment, our approach goes beyond linearity, and the individual, structural and organisational level for the study of Athena SWAN interventions, aiming to address them in their context and in relation to emerging conditions. In conclusion, we study the Athena SWAN Charter having as a point of departure the following parameters: (1) the complexity of design and implementation and the numerous interacting factors that are context dependent and produce complex settings; (2) the non-linearity of the interventions and the constantly emerging conditions; and (3) the notion that impact in dynamic, complex contexts is considered in terms of contribution – not attribution – and the probability of the programme design to foster change [46].

## Methods

### Study aim and objectives

The aim of this study is to analyse Athena SWAN Silver action plans in the medical sciences at Oxford University, using a complexity approach and a comparative European perspective based on the typology of gender equality interventions developed by the Horizon 2020 project Evaluation Framework for Promoting Gender Equality in Research and Innovation (EFFORTI) [46]. The methodology is hence a case study with additional comparative dimensions. The objectives of the study are as follows:

- To explore what types of interventions are associated with Athena SWAN Silver action plans.
- To compare these types of interventions with those used in the wider European Research Area.
- To discuss how a complexity approach can provide insights for policy and practice.

### Study setting and context

The study is set within the context of the medical sciences at the University of Oxford. The university tops the Times Higher World University Rankings [47], outperforms other universities in EU research funding competitions [48], and files more international patent applications to the World Intellectual Property Organization than any other university in Europe [49]. To ensure its competitive advantage, the university is actively seeking new ways to attract the best students, recruit and retain the most talented staff, and increase research funding. Thus, advancing gender equality in education, research and innovation is one of the key strategic priorities for the university [14, 50–52]. It has been a member of the Athena SWAN Charter since its establishment in 2005 and, as of October 2018, holds 19 Silver and 15 Bronze departmental level awards in medical sciences, mathematics, physics, life sciences and social sciences. The overwhelming majority (16/19) of Silver awards are in medical sciences, reflecting the linkage of Athena SWAN Silver awards to NIHR competitive biomedical research funding.

### Data collection and analysis

The study is based on the document analysis of all 16 departmental Athena SWAN Silver action plans (1245 pages) in medical sciences at the University of Oxford. We collected the required action plans from a dedicated university webpage, where all successful award applications and action plans were publicly available [53]. We extracted data pertinent to the design and implementation of gender equity interventions, classified the target population of each action by gender and staff category, analysed these data thematically [54], and coded the emerging themes in Microsoft® Office using categories from the 2015 Athena SWAN Charter Awards Handbook [55]. In doing so, we were sensitised by the concepts pertaining to complexity theory, i.e. a systemic approach, multi-layered context, target populations, logic of change, feedback loops, dynamic interaction, emergent causality and adaptation to changes. We used the process of constant comparison to analyse emerging themes in a comparative European perspective. We reached consensus on themes and comparison by agreement. We also reflected on our own prior views and experiences, which may have influenced our analysis and interpretation of data.

## Results

**Fig. 1:**
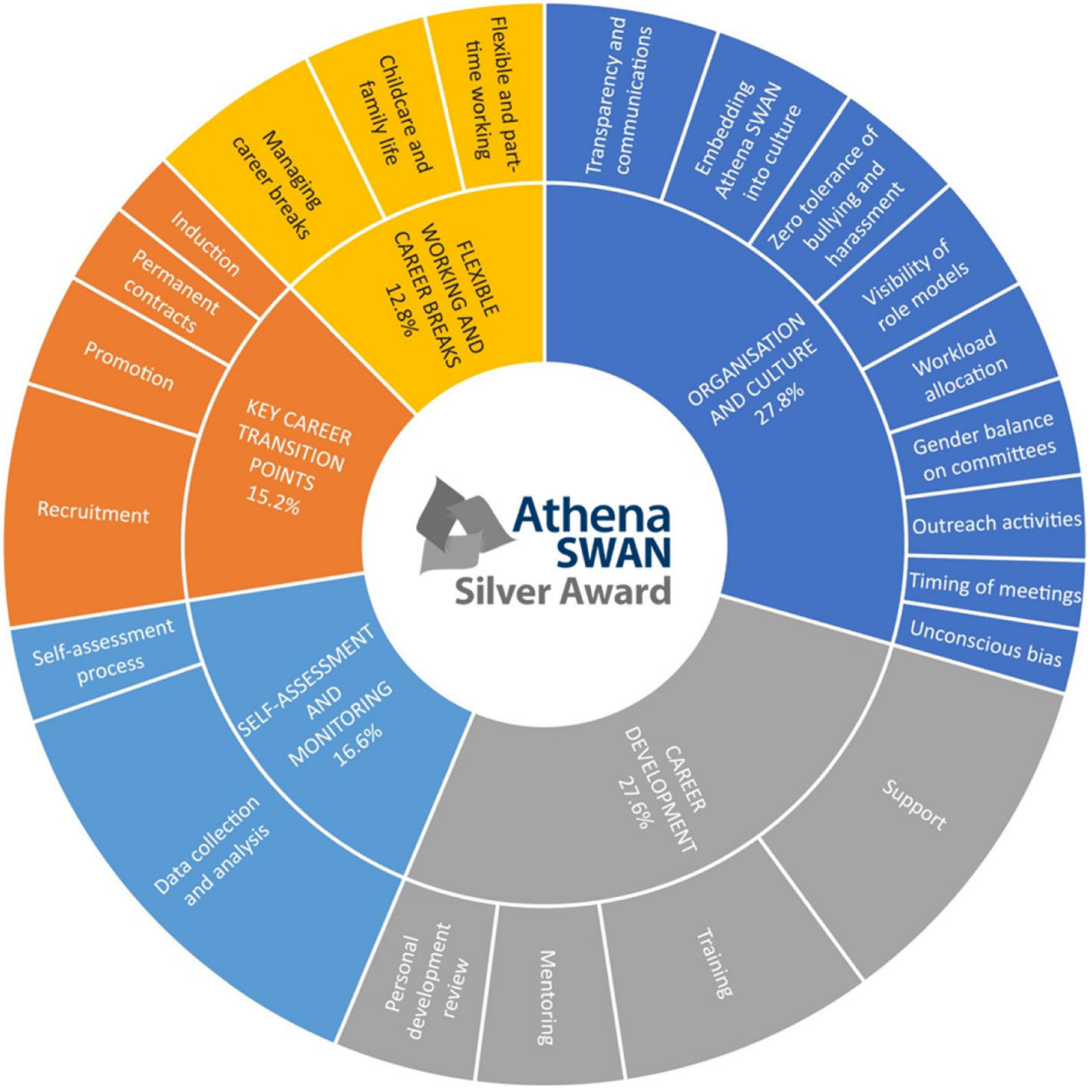
Athena SWAN Silver award interventions by theme, subtheme and frequency of actions in 16 departmental action plans in medical sciences at the University of Oxford, 2014–2017

In total, 16 Athena SWAN Silver action plans contained 547 actions pertaining to gender equality interventions. On average, there were 34 actions per action plan. The target population of the Athena SWAN Silver actions analysed by gender were all genders indiscriminately (88%), women (11%) and men (1%). The target population of the Athena SWAN Silver actions analysed by student and staff category were academic and research staff (52%), all staff (32%), students (1%), students and staff (4%), and professional and support staff (1%). Thematic analysis of actions against the relevant sections and subsections of the 2015 Athena SWAN Charter Awards Handbook [55] resulted in five themes and 22 subthemes. According to frequency analysis of actions by theme, ‘Organisation and culture’ (28%) and ‘Career development’ (28%) were the most frequent themes, followed by ‘Self-assessment and monitoring’ (17%), ‘Key career transition points’ (15%), and ‘Flexible working and career breaks’ (13%). Themes and subthemes are summarised and visually represented in Fig. 1 using the sunburst chart technique, whereby colour-coded concentric circles display a hierarchical relationship between major themes and subthemes in proportion to the frequency of actions pertaining to each theme and subtheme.

A comparison of the SWAN Silver action plans with the EFFORTI typology of gender equality interventions in research and innovation revealed that they represent 78% (31/40) of gender equality intervention types used in the wider European Research Area and a further 8 distinctive intervention types (Table 1). Gender equality interventions in the wider European Research Area tend to target primarily women in academic and research roles to address their underrepresentation in European research organisations through a range of interventions, including, among others, affirmative action such as quotas, funding and positions reserved to women. Athena SWAN has a somewhat broader focus as regards the target population, including also professional and support staff and students as well as considering the intersectionality of gender and other aspects of identity, such as sexuality, race, disability, age and religion. Yet, Athena SWAN lacks the intervention types based on the wider European Research Area objective of integrating the gender dimension in research and education (Table 1).

**Table 1:** Comparison of gender equality interventions distinctive to Athena SWAN and EFFORTI

	Gender equality interventions distinctive to Athena SWAN	Gender equality interventions distinctive to EFFORTI
Organisational, structural and cultural actions	1. Institutionalised self-assessment teams 2. Revising timing of meetings and events 3. Workload allocation model 4. Mandatory training on unconscious bias and bullying and harassment	1. Institution of quotas 2. Introduction of chairs and positions reserved for women 3. Special funding for women researchers
Career development	1. Career development interventions targeting professional and support staff 2. Career development interventions targeting students 1. Addressing a full continuum of key career transition points	2. Support of mobility, including spouse relocation schemes
Assessment and monitoring	1. Intersectional approaches to data collection and analysis	
Integration of the gender dimension in research and education		1. Integration of the gender dimension and impact in research 2. Integrating the gender dimension in tertiary education 3. Revision of teaching curricula and texts 4. Introduction of single-sex degree and specialisation courses 5. Provision of gender and women’s studies or modules

In what follows, themes and subthemes of the analysed actions are presented in the order of appearance in the Athena SWAN Silver award application, together with 93 illustrative examples of actions. Illustrative actions are intended to provide researchers and practitioners with a compact overview of the range of actions and how they are formulated in the actual action plans. Given that publicly available Athena SWAN action plans assume all actions being equal, illustrative actions are presented in no particular order of importance or priority. Each illustrative action contains the code corresponding to the name of the department and the numbering of actions in the relevant action plan as well as the description of the target population by staff category and gender. The names of departments and their codes are presented in Additional file 2. The departmental Silver award applications and action plans analysed during the current study are provided in Additional file 3.

### Self-assessment and monitoring

#### Self-assessment process

To participate in the Athena SWAN Charter, every department must have established a self-assessment team (SAT) with broad representation of people and skills across the department and the remit to reflect on quantitative and qualitative data on gender equity, evaluate relevant policies and practices, establish priority areas and targets, develop an evidence-based action plan, and evaluate its effectiveness against the agreed objectives [55]. Specific actions regarding self-assessment focus on institutionalising the SAT and its working groups within the departmental structure and securing necessary leadership and administrative support.

- “*The SAT will develop and publish terms of reference including guidelines on purpose, recruitment to the committee, roles, length of service and will embrace a vision of committee aims*.” (D1, 2, target population: all categories of staff of all genders)
- “*Embed work of the Athena SWAN Career Development Working Group in permanent training and development infrastructure of the* [department].” (D16, 6.3, target population: all categories of staff of all genders)
- “*SAT will meet termly to discuss the implementation and progress of the Silver action plan*” (D15, 1, target population: all categories of staff of all genders)
- “*Explore appointment of a new post, an Athena SWAN lead, to the administrative team*.” (D8, 1.1, target population: all categories of staff of all genders)

#### Data collection and analysis

Departmental SATs use a variety of sources, including surveys, focus groups, interviews and databases, to collect and analyse data on gender equality among staff and students at all levels. Increasingly, SATs employ intersectional approaches to better understand the issues at the intersection of gender and other aspects of identity such as sexuality, race, disability, age and religion [56]. Although there is a growing appreciation of the importance to consider gender fluidity and non-binary gender factors, action plans address gender equity predominantly in binary terms. Actions to improve data collection and analysis range from making it more regular, reaching out to broader student and staff populations, and refining existing questions to trialling new methods, carrying out new types of analysis, and investigating new questions.

- “*The SAT will run regular staff/student surveys and convene focus groups to assess the impact of our action plan*” (D4, S1.2, target population: all categories of staff of all genders)
- “*The question to determine gender in the survey should be posed as: ‘Female, male, self-defined, or prefer not to say’ to capture the full spectrum of gender identities*.” (D5, 1.4, target population: all categories of staff of all genders)
- “*Trial exit interviews to determine if they are an efficient way of capturing necessary information (balance of staff time vs. quality of information gathered)*.” (D5, 2.4, target population: all categories of staff of all genders)
- “*Carry out a pay audit of all staff by grade scale point and gender*.” (D13, 6.9, target population: academic and research staff of all genders)
- “*Investigate the barriers for appointment to senior clinical posts overseas*.” (D10, 4.4, target population: academic and research staff of all genders)

### Key career transition points

The Athena SWAN self-assessment process challenges departments to develop and improve policies and practices to help different categories of staff to understand and navigate through key career transition points, from recruitment into a new role to induction to a new workplace, promotion to a new position, and securing a permanent or long-term contract of employment. The latter is particularly important in the given research-intensive setting characterised by a high proportion of staff of all genders in medical sciences departments on fixed-term and open-ended contracts (80%) and gender disparity in permanent employment (15% female vs. 26% male) [20].

#### Recruitment

Departments strive to improve their recruitment practices by developing workforce intelligence and planning, improving the attractiveness of job opportunities to female applicants, using targeted recruitment as well as by minimising selection bias through mandatory training, gender balance on selection panels and more inclusive decision-making.

- “*Investigate recruitment and subsequent working experience of members of minority groups working in the* [department].” (D16, 2.2, target population: all categories of staff of all genders)
- “*Identify skills shortages and underrepresentation in* [research] *groups; establish future staffing requirements and succession plans; present to* [the Equality Committee] *as a ‘Workforce Plan’*.” (D12, 1.1, target population: academic and research staff of all genders)
- “*Encourage more female applicants by highlighting that we will provide assistance when applying for nursery/childcare/school places*.” (D4, S3.4, target population: female academic and research staff)
- “*Implement the new Electoral Board process for appointment of Statutory Professors and introduce equivalent* [departmental] *process for all other senior posts, and pilot the use of head-hunters*.” (D13, 1.2, target population: academic and research staff of all genders)
- “*Ensure that all people involved in recruiting (not just panel chairs) have completed recruitment and selection training*.” (D5, 3.3, target population: academic and research staff of all genders)

#### Induction

Departments work to enhance the quality of their induction programmes, dedicated webpages, factsheets and other materials for new recruits by tailoring them to different career stages, sites and research groups, introducing networking and peer-support schemes as well as monitoring their effectiveness and checking the awareness of key policies, resources and career development opportunities.

- “*Develop a tailored induction programme for senior researchers, group leaders and line managers with emphasis online management responsibilities and the department’s family-friendly culture*.” (D13, 2.1, target population: academic and research staff of all genders)
- “*Develop with the newly formed Postdoctoral Society a ‘Buddying’ support system for new postdocs joining the department*” (D4, S4.3, target population: academic and research staff of all genders)
- “*HR to hold an induction/probationary meeting 3 months after the start date to ensure that the new starter is feeling settled and to check they are aware of policies, postdoc events, etc*.” (D5, 3.7, target population: all categories of staff of all genders)
- “*Ensure initial career development discussions are held during probation*.” (D10, 3.1, target population: academic and research staff of all genders)
- “*Continue to monitor the effectiveness of the induction process, and identify areas for improvement. Develop a mechanism to monitor the effectiveness of site-specific inductions*.” (D15, 10, target population: academic and research staff of all genders).

#### Promotion

Departmental action plans are in place to accelerate the career advancement of all eligible staff through the existing regrading, recognition of distinction and award schemes. A range of actions includes raising awareness and transparency of promotion opportunities, conducting gender-sensitive review of promotion criteria and salaries, identifying and encouraging all eligible candidates, and women specifically, to apply for promotion.

- “*For non-clinical academics, transparency of pay rises, promotions process and equivalency of tenure tracks need to be continuously developed*.” (D9, S8, target population: academic and research staff of all genders).
- “*Annual review of salaries to ensure parity and gender balance*.” (D8, 3.4, target population: academic and research staff of all genders)
- “*Continue to promote and develop criteria for prizes to ensure they are achievable for both men and women; actively show how women have met the criteria and provide case studies*.” (D10, 2.5, target population: students of all genders)
- “*An annual audit will be conducted via* [a university publications management system], *of peer reviewed publications first authored by* [early and mid-career researchers], *taking account of part time work and family leave*.” (D11, 2.3, target population: academic and research staff of all genders)
- “*Continue to identify women and provide administrative support for promotion applications through* [Recognition of Distinction] *award for Professorships, Associate Professorships and University Research Lecturer scheme*.” (D12 1.5, target population: female academic and research staff)

### Permanent and long-term contracts

Athena SWAN has spurred departments in medical sciences to provide more job security for academic-related and research staff, the overwhelming majority of whom compete for research funding in a tough market and remain on short fixed-term contracts. Departments take action to transfer eligible staff on to open-ended contracts with support for at least as long as external research funding is available and set targets to increase the number of staff, especially women, on permanent contracts:

- “*Implement a transparent Department wide policy to review all staff on fixed-term contracts on a regular basis. Move staff from fixed-term to open contracts, where possible*.” (D8, 3.2, target population: all categories of staff of all genders)
- “*We will introduce a clear and transparent system to allow the transfer of senior research fellows on to permanent contracts*.” (D4, S5.1, target population: academic and research staff of all genders)
- “*Increase the proportion of Associate Professor and Full Professorial posts that are held by women from the current 38.7% (12/31) to 50% by 2018*.” (D2, 5.3, target population: female academic and research staff)
- “*Investigate mechanisms underlying high attrition rate of female academic clinicians*.” (D14, 3.5, target population: female academic and research staff)

### Career development

In addition to improving policies and practices for understanding and navigating through key career transition points, the Athena SWAN self-assessment process challenges departments to provide staff with career development opportunities. These include acquiring new skills through training, periodically reviewing career objectives through personal development reviews, mentoring by colleagues at more advanced career stages, and professional and peer support.

#### Training

Departments seek to promote career development not only of academic and research staff but also of professional and support staff and students through training, including courses specifically designed for women. Actions are in place to better identify the training needs of particular groups of staff and provide more in-house and external training with regards to management, leadership and negotiation skills, career planning, and grant writing.

- “*Encourage management training (appraisals, project management, coaching, time management, and workload planning) for Principal Investigators, supervisors and line managers*.” (D10, 3.2, target population: academic and research staff of all genders)
- “*Organise targeted ‘How to’ workshops designed to help staff at the key career transition points (e.g. writing a grant application).*” (D13, 1.9, target population: academic and research staff of all genders)
- “*Identify senior staff, and those approaching senior grades, who are seeking training in ‘leadership’ from the* [personal development review] *discussion and provide 5* [departmental] *funded places (up to £5000 per place) on a leadership course for senior women.*” (D8, 4.4, target population: academic and research staff of all genders)
- “*Organise a ‘Manage Your Supervisor’ training session during May for first year DPhil students.*” (D13, 5.1, students of all genders)

#### Personal development review

Athena SWAN has been instrumental to the institutionalisation of annual personal development review (PDR), which enables staff to have open conversations with their reviewer about their role, career aspirations and development opportunities. Departments work to increase the awareness and uptake of PDR, provide training and guidance for reviewers and reviewees, and improve its effectiveness, especially for researchers on a succession of short-term contracts, postdocs approaching independence and other staff for whom PDR is particularly beneficial.

- “*From 2017 we will move to undertaking PDR annually in April/May for all staff. We will provide flexibility for clinicians who would prefer a different time of year to enable their University PDR to inform their* [National Health Service] *appraisal*.” (D11, 9.1, target population: all categories of staff of all genders)
- “*Continue to reinforce to staff the necessity to follow-up on the PDR discussions periodically during the year in order to ensure progress towards the training and personal development goals*.” (D12, 2.7, target population: academic and research staff of all genders)
- “*Annual PDR workshops for staff, which covers the purpose of PDR, guidance on the conduct of PDR and the rationale for why the Department aims to increase uptake*.” (D8, 4.2, target population: academic and research staff of all genders)
- “*Add a checklist to the PDR form to encourage discussion of scientific engagement, internal and external mentoring programmes, eligibility and suitability for recognition of distinction, committee membership and external positions of influence*.” (D5, 4.2, target population: academic and research staff of all genders)

#### Mentoring

Due to Athena SWAN, all departments have already established formal mentoring schemes for academic and research staff. Current actions aim to increase the uptake of mentoring, extend formal mentoring schemes to include all categories of staff and students, provide training in effective mentorship, and trial new approaches. In addition to mentoring, which provides mentees with career advice by peers at more advanced career stages, some departments are establishing sponsorship schemes, whereby members of staff are paired with senior level members of staff who have a vested interest in their career success and advocate on their behalf.

- “*Keep encouraging postdocs and early career researchers to join the established* [mentoring] *scheme by publicising its benefits on our website and bulletin*.” (D9, S16A, target population: academic and research staff of all genders)
- “*Improve opportunities for mentoring, particularly for professional and support staff*.” (D16, 3.6, target population: professional and support staff of all genders)
- “*Provide training in effective mentorship to all managers*.” (D12, 2.16, target population: academic and research staff of all genders)
- “*Trial a scheme where junior clinical staff are assigned a senior sponsor who will be their advocate, including in the NHS clinical setting where the working environment can be challenging.*” (D13, 1.15 target population: academic and research staff of all genders)

#### Professional and peer support

Departments organise workshops, coffee mornings, peer support groups and subject-specific events to help students and early career researches as well as professional and support staff to make informed career choices. The main focus of support with career development of academic and research staff is on securing external research funding and establishing independence. Departments provide methodological training, administrative support and internal peer review, and help develop interview skills for fellowship and grant applications, commit internal funding and support of senior researchers to develop applications, and seek to increase teaching opportunities for junior researchers.

- “*Provide a fellowship coordination process to ensure all applications receive the same support (e.g. internal review, mock interview)*.” (D13, 1.12, target population: academic and research staff of all genders)
- “*Develop a mechanism for staff to ‘bid’ for funded protected time to work on fellowship and grant applications*.” (D8, 5.2, target population: academic and research staff of all genders)
- “*Encourage senior staff to provide junior researchers with the opportunity to be a co-applicant on grant applications*.” (D8, 5.3, target population: academic and research staff of all genders)
- “*Generate greater teaching opportunities for junior researchers, and monitor gender balance of uptake*.” (D15, 16, target population: academic and research staff of all genders)
- “*Use Autumn School* [for clinical medical students and foundation doctors] *as a vehicle to inspire potential female academic psychiatrists*.” (D14, 3.7, target population: female students)

### Flexible working and managing career breaks

#### Flexible and part-time working

Athena SWAN helps to improve arrangements for flexible and part-time working for all genders and groups of staff. Departmental action plans include interventions to promote the value of flexible and part-time working, raise awareness about the existing arrangements, formalise them through policies and guidelines as well as extend to graduate research students.

- “*Continue to promote and de-stigmatise the value of flexible working and clarify the process for requesting this. Encourage culture of monitoring output rather than ‘presenteeism’*.” (D10, 1.7, target population: all categories of staff of all genders)
- “*Raise awareness of the flexible working policy: 26% of staff do not know about the flexible/part-time working policy*.” (D2, 6.2, target population: all categories of staff of all genders)
- “*Create guidelines for staff and their line managers explaining what part-time working entails and what to consider when deciding whether or not to become a part-time member of staff*.” (D11, 8.6, target population: all categories of staff of all genders)
- “*Explore opportunities for part-time work in* [departmental] *Clinical Research Facilities to facilitate career re-entry for clinicians*.” (D13, 1.16, target population: academic and research staff of all genders)
- “*Include part-time DPhil in graduate advertising, target clinical academic mentors to advertise the programme and explore sources of funding*.” (D8, 2.3, target population: students of all genders)

#### Managing career breaks

Athena SWAN has prompted departments to provide more support to women with managing maternity and other career breaks as well as to introduce paternity and shared parental leave policies aimed at men. Departmental action plans contain further actions to improve the implementation of the existing policies and to commit resources to helping academic and research staff to return to research following a career break or a period of leave for caring responsibilities.

- “*Plan how Shared Parental Leave will be managed in the department; also how to encourage women to consider sharing leave with their partner, and men to take leave*.” (D5, 7.1, target population: men in all categories of staff)
- “*Introduce ‘Buddy System’ for staff on maternity, paternity, caring or sick leave to help ensure that people are kept up-to-date with departmental decisions and policies*.” (D2, 6.3, target population: all categories of staff of all genders)
- “*Women returning to work after a period of maternity leave are to be given dispensation from teaching commitments*.” (D9, S23a, target population: female academic and research staff)
- “*Continue to promote the Returning Carers’ Fund and encourage and support applications*.” (D12, 5.1, target population: academic and research staff of all genders)

#### Childcare and family life

Departments work to enhance the provision of childcare in their specific locations and help staff to reconcile work and family life more broadly. Many departments improve information about available childcare services, invest into the provision of parking, breastfeeding facilities and sponsored nursery places, and try to create a more family-friendly environment.

- “*Provide pregnancy car parking space for expectant mothers who are finding their usual mode of transport to work challenging*.” (D5, 7.6, women in all categories of staff)
- “*Our maternity/paternity focus group meeting raised the issue of a lack of breastfeeding support and facilities in the Department and the provision of a private room for breastfeeding is now underway*.” (D4, S6.4, women in all categories of staff)
- “*Invest in sponsored nursery places*.” (D2, 6.5, target population: all categories of staff of all genders)
- “*Improve environment for women to discuss issues of home/work-life balance with their line manager/supervisor*.” (D7, 11, women in all categories of staff)
- “*Support family friendly events in the divisions, to bring together staff, students and their families, and foster a sense of community in the department.*” (D13, 4.6, target population: all categories of staff of all genders)

### Organisation and culture

#### Embedding principles of Athena SWAN into culture

Departments actively seek to embed the principles promoted by Athena SWAN into key processes and decision-making points by considering equality, diversity and inclusion as part of their values and identity, norms and procedures, social events, and working environments.

- “*Design a set of core values to reflect the ethos of the Department; gain approval from* [the Executive Committee]*; publish on website*.” (D12, 4.8, target population: all categories of staff of all genders)
- “*Ensure diversity in imagery used on our website, in publicity materials and in social media*.” (D16, 5.3, target population: all categories of staff of all genders)
- “*Highlight Athena SWAN in all job advertisements*.” (D10, 3.5, target population: academic and research staff of all genders)
- “*Identify suitable speakers from outside the department to run a workshop for* [group leaders] *on how to create a positive and supportive culture in the lab*.” (D12 2.14, target population: academic and research staff of all genders)
- “*Make changes to working environments to improve the quality of working life*.” (D3, 4.1, target population: all categories of staff of all genders)

#### Transparency and communications

Many actions to promote equality and inclusion focus on improving the transparency of departmental structures and decision-making, enabling equal access to key people and resources, and diversifying internal communication strategies.

- “*Transparency, particularly regarding management structures and decision-making, will be further improved using a variety of communication strategies*.” (D1, 17, target population: all categories of staff of all genders)
- “*Set up a new sharepoint site for minutes of all meetings. Notify staff through the Weekly News that minutes have been published. Use multiple methods to give feedback on key issues including summarizing decisions in the department newsletter and at the termly Department open meeting*.” (D11, 7.1, target population: all categories of staff of all genders)
- “*Create admin postcards to distribute at admin surgeries to illustrate pipelines and contact persons for different processes (e.g. applying for a grant, recruiting a new staff member)*.” (D6, 5.2, target population: academic and research staff of all genders)
- “*Ensure greater acknowledgement of success and achievement by adding success stories to the* [Departmental] *Digest and display screens in reception*.” (D4, S5.10, target population: academic and research staff of all genders)

#### Zero tolerance of bullying and harassment

The Athena SWAN process provides departments with the opportunity to strengthen their core human resource policies to create a more positive culture for everyone. Most notably, all departments strive to eradicate bullying and harassment from the workplace by raising awareness of zero tolerance of bullying and harassment and providing resources to address it.

- “*Reinforce messages about zero tolerance of bullying and harassment*.” (D16, 5.2, target population: all categories of staff of all genders)
- “*Initiate annual email to all postdocs reminding them of the bullying and harassment policy and the help available to raise awareness following the survey results*.” (D5, 8.2, target population: academic and research staff of all genders)
- “*We will include details of the Departmental bullying and harassment officers on the posters of key people and add this information to the intranet*.” (D4, S5.7, target population: all categories of staff of all genders)
- “*Appoint an external independent mediator/listener to investigate the nature and extent of the problem (e.g. through targeted mini-survey)*.” (D13, 3.6, target population: all categories of staff of all genders)
- “*Continue to provide a programme of in-house training on Bullying and Harassment*.” (D8, 7.2, target population: all categories of staff of all genders)

#### Mandatory unconscious bias training

Another important human resources policy that many departments introduce as part of the Athena SWAN process is mandatory unconscious bias training. In addition to online training provided by the University, departments develop in-house online and in-person training and ensure completion by staff and students.

- “*Introduce mandatory online equality & diversity and unconscious bias training for all staff and students*.” (D13, 3.1, target population: all categories of staff and students of all genders)
- “*Offer in-house unconscious bias training annually. Monitor compliance with compulsory training requirements. Include training records in staff database to check compulsory requirements*.” (D6, 4.12, target population: academic and research staff of all genders)
- “*We will chase up the 5% of group leaders who did not attend the unconscious bias training to complete an online version of the course to ensure 100% compliance*.” (D5, 3.6, target population: academic and research staff of all genders)

#### Gender balance on committees

All departments work towards improving gender balance on committees but with a varying degree of ambition. Whereas some departments aim to improve gender balance relative to the proportion of group leaders, others open up membership to all students and staff aiming to achieve absolute gender balance.

- “*As number of female group leaders increases, increase their participation on committees. Aim to keep slightly ahead of simple proportion of female group leaders*.” (D5, 6.2, target population: female academic and research staff)
- “*Review the membership of* [Departmental] *committees and identify more women as potential members (opening up membership of committees to students,* [postdoctoral research assistants] *and support staff where appropriate)*.” (D13, 3.4, women in all categories of staff and students)
- “*Ensure committees are gender-balanced. Monitor committee membership and attendance records. Rotate membership and chairs, with future vacancies appointed by advertisement and election. Monitor the reasons for requests to opt-out of committee membership*.” (D15, 25, target population: academic and research staff of all genders)
- “*Achieve gender balance on departmental committees*.” (D14, 5.4, target population: academic and research staff of all genders)

#### Workload allocation model

The Athena SWAN process challenges departments to develop a fair and transparent workload allocation model, monitor it for gender bias, and use it for personal development review and promotion. Different departments are at different stages of implementing such a model and, in the majority of departments, it remains limited to academic and research staff.

- “*Set up a process, initially within PDR to look at workload, i.e. time spent on different core activities (research, internal administration and management committees, teaching and supervision, and outreach activities)*.” (D8, 4.3, target population: academic and research staff of all genders)
- “*Work with* [the Medical Sciences Division] *to design a workload allocation model for clinical departments*.” (D12, 4.9, target population: academic and research staff of all genders)
- “*The workload model analysis will be continued on an annual basis to work towards parity between male and female group leaders. We will publicise our workload model within the University by arranging a workshop with other Departments in* [Medical Sciences] *and other Divisions to discuss best practice. This will also feed into refining our model*.” (D1, 16, target population: academic and research staff of all genders)
- “*Increase the proportion of staff who feel that their workload allocation is fair. Increase the transparency of workloads*.” (D2, 5.4, target population: academic and research staff of all genders)

#### Timing of departmental meetings

As part of Athena SWAN bronze awards, the majority of departments have addressed the timing of departmental meetings and social events to make them possible to attend for staff with caring responsibilities and working part-time. Several departments, especially clinical ones, continue to reinforce the importance of inclusivity for all meetings and events.

- “*Ensure that there is a high level of awareness of the concept of core hours by including it in staff induction material and in the monthly newsletter*.” (D5, 7.8, target population: academic and research staff of all genders)
- “*Promote inclusive meeting etiquette*.” (D10, 5.2, target population: academic and research staff of all genders)
- “*Schedule departmental meetings and seminars between 9.30am – 2.30pm wherever possible, and give considerable notice ahead of all day and evening events*” (D13 4.3, target population: all categories of staff of all genders)
- “*As far as practical, reduce scheduling of key meetings in school holidays*.” (D5, 7.7, target population: academic and research staff of all genders)

#### Visibility of role models

All departments take action to promote the visibility of role models and build gender equality and diversity into the organisation of events and online materials.

- “*Change the way the external seminar series is organised so that all potential seminar hosts have to nominate 2 speakers; 1 female and 1 male*.” (D12, 4.3, target population: academic and research staff of all genders)
- “*Increase the number of female research staff with a personal webpage*.” (D4, S3.10, target population: female academic and research staff)
- “*Linking to the Staff Profiles area on the department website provide example career trajectories from* [professional and support staff] *at different points in their careers*.” (D11, 5.1, target population: professional and support staff of all genders)
- “*We are currently developing a website based on digital video interviews with university staff with a range of disabilities*.” (D11, 10.2, target population: all categories of staff of all genders)

#### Outreach activities

The Athena SWAN process recognises the value of public engagement with science. This encourages departments to improve and reward outreach activities.

- “*Improve profile of public engagement section of website and encourage people to submit examples of outreach to go on the News section and on display screens in reception*.” (D4, S5.8, target population: all categories of staff of all genders)
- “*Encourage more male researchers to take up Comms training and get involved in public engagement activities in the department – actively seek men to take part*.” (D11, 8.3, target population: male academic and research staff)
- “*Run a series of public engagement/school outreach activities to attract female applicants from physical science disciplines, in which women are traditionally underrepresented*.” (D12, 1.2, target population: female academic and research staff)

### Complex contextually embedded system of dynamic action planning

**Fig. 2:**
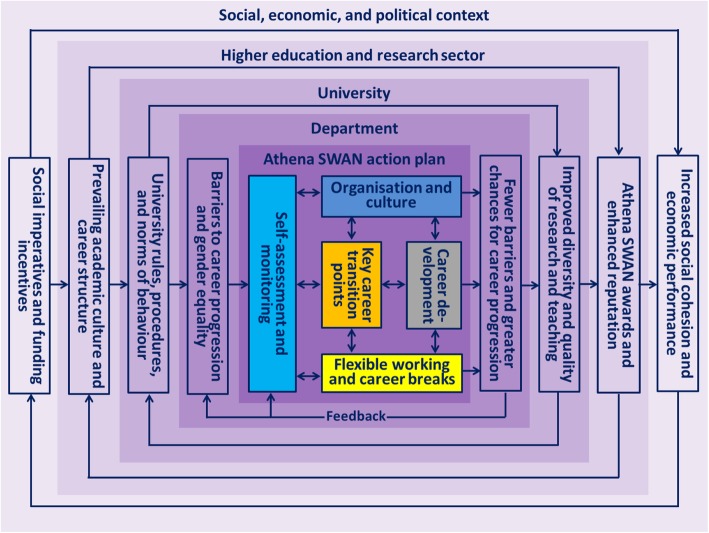
A complex contextually embedded system of Athena SWAN’s dynamic action planning

Figure 2 synthesises and visually represents the analysis of Athena SWAN Silver action plans pertaining to complexity. Namely, Fig. 2 demonstrates that the five types of actions described above are organised into a complex contextually embedded system of dynamic action planning and tailor-made to challenge gendered barriers to career progression at multiple, interacting levels. The systemic approach embraced herewith allows us to take a holistic view, considering that all parts of the system are interlinked. We thus consider the complex system in which the Athena SWAN scheme operates, acknowledging its non-linear character and the multitude of variables at play. In what follows, we narratively synthesise our findings around two major themes that emerged from our analysis, namely (1) dynamic linkage of departmental action plans to a multi-layered context and (2) system-level organisation with positive feedback loops and new emergent properties.

#### Dynamic linkage of departmental action plans to a multi-layered context

Departmental action plans are dynamically linked to the wider social, economic and political context, the higher education and research sector, and the university, which constitute a complex system. The widespread development and implementation of Athena SWAN Silver action plans resulted from the changes in the social, political and economic context. Namely, the social progress imperative to improve gender equity in biomedical research prompted the government Department of Health to introduce NIHR funding incentives, in response to which the university and departments developed and implemented Athena SWAN Bronze action plans.

Following positive feedback in the form of an Athena SWAN Silver award, departments have developed and now implement Athena SWAN Silver action plans. They build on the outcomes of the previous Athena SWAN Bronze action plans, comments from the Athena SWAN peer review panel, and emerging best practice from the network of Athena SWAN Charter members representing the wider higher education and research sector. The latter is particularly important because the prevailing academic culture and career structure influences the range of possibilities for change within individual universities. Hence, collective efforts of the entire higher education and research sector are often required to enable changes within individual universities. The context of individual universities is equally important because university rules, procedures, and both formal and informal norms of behaviour shape the range of interventions that departments can implement to remove barriers to career progression and gender equality within departments.

#### A system-level organisation with positive feedback loops and new emergent properties

Interactions among the five types of actions in departmental action plans create a system-level organisation with positive feedback loops and new emergent properties. Firstly, departmental self-assessment teams continuously assess data and evaluate the implementation of actions and their short- and medium-term impact. In response to the changing contextual factors and emerging evidence, they adapt on-going actions and develop new ones. In doing so, actions aimed at self-assessment and monitoring not only determine which actions are included in the action plan but also regulate their implementation. In a complex system such as Athena SWAN, the agents of change are interconnected and affect each other. Thus, small alterations initiated by the self-assessment team can lead to larger effects at a later point in time as, at critical points, small changes may have great impact [37]. Impact is often indirect and long-term [57]. The production of impact is closely connected to the ability of a programme to foster the right conditions for change. This implies that increased probability of change may be part of the expected impact of complex interventions [58]. In line with the distinction made between complicated interventions, which have many but foreseen components, and the Athena SWAN as a complex intervention – characterised by non-linearity, uncertainty and emergence – the expected impact of Athena SWAN needs to be considered in terms of how the programme fosters conditions for change and increases the probability that change can occur in the particular context of the medical sciences departments [35].

Secondly, given that organisation and culture enable and constrain all interactions in the department, actions aimed at changing departmental organisation and culture also influence the other types of actions that adapt in response to the changing organisation and culture. There are multiple choices to make for staff and students that are subject to a range of contextual conditions, structural resistances and other constraints that impact career progression.

Thirdly, actions aimed at flexible working and managing career breaks also dynamically interact with the other types of actions, in particular, with key career transition points and career development opportunities for staff and students taking advantages of flexible working arrangements and those taking career breaks. In complex systems, actions of different agents of change, such as individual choices of staff and students in terms of training activities, careers, courses, etc., can lead to increased gender equality in the long run.

Finally, as more career development opportunities and better conditions for career progression through key career transition points emerge as a result of the implementation of action plans, key career transition points come faster and the chances of progressing to the next career stage in the new emergent conditions increase. Moreover, in contrast to complicated systems, complex systems are adaptive, which in this particular context means that they respond to the changes initiated through Athena SWAN. In this respect, Athena SWAN action plans adapt to constantly moving targets and consider new emergent conditions.

### Comparative European perspective – strategic opportunities to strengthen gender equality policies in the wider European Research Area

In order to further explore the strengths and opportunities to expand the scheme, we have analysed the Athena SWAN interventions in a comparative European perspective based on a typology of gender equality interventions in research and innovation developed within the EU Horizon 2020 project EFFORTI [59]. As pointed out above, this is not a full-fledged comparative analysis but rather a comparison of the Athena SWAN scheme with the state of the art regarding types of gender equality interventions, generated in the frame of the EFFORTI project. Initially proposed by Kalpazidou Schmidt and Cacace [35], based on an empirical study of 109 gender equality interventions worldwide, and a literature review [59], the EFFORTI typology of gender equality interventions in research and innovation was subsequently adapted and expanded to synthesise knowledge on gender equality interventions from major European projects such as GEAR (Gender Equality in Academia and Research) [33], GEDII (Gender Diversity Impact – Improving research and innovation through gender diversity) [60], GENERA (Gender Equality Network in the European Research Area) [61], Gender-NET (Promoting Gender Equality in Research Institutions and Integration of the Gender Dimension in Research Content) [62], PRAGES (Practicing Gender Equality in Science) [63], and STAGES (Structural Transformation to Achieve Gender Equality in Science) [64].^3^ An overview of the EFFORTI typology of gender equality interventions in research and innovation has been elaborated elsewhere [46] and is summarised in Additional file 1. The comparison of the Athena SWAN Silver gender equality action plans with the EFFORTI typology of gender equality interventions in research and innovation shows that Athena SWAN is the single most comprehensive and inclusive gender equality scheme in Europe. Athena SWAN covers approximately three-quarters of gender equality intervention types used in the wider European Research Area (Fig. 1 and Additional file 1). Furthermore, there are some gender equality interventions distinctive to Athena SWAN (Table 1). While gender equality interventions in the European Research Area tend to focus primarily on women in academic and research roles, Athena SWAN has a broader focus on all categories of staff and students, predominantly, regardless of their gender, taking into account considerations of intersectionality such as sexuality, race, disability, age and religion.

Athena SWAN also has a more contextually embedded, country-wide systemic approach to action planning than any other single gender equality scheme in Europe, especially with regard to system-level interventions related to institutionalised SATs, considerations of intersectionality, key career transition points, career development, mandatory training on unconscious bias and bullying and harassment, timing of meeting and events, and a workload allocation model. Gender equality policies in the wider European Research Area can benefit from exploring Athena SWAN’s contextually embedded systemic approach to dynamic action planning and inclusive focus on all genders and categories of staff and students. Yet, Athena SWAN has two limitations with regard to intervention types. Whereas some European countries intervene to introduce quotas, chairs and positions reserved to women, funding for female researchers, and single-sex degree and specialisation courses, Athena SWAN does not promote such interventions because, under the United Kingdom Equality Act 2010, they may be interpreted as positive discrimination and therefore deemed unlawful. Moreover, Athena SWAN misses the opportunity to promote the integration of sex and gender dimension in research and education, which is particularly important both in the wider European Research Area and globally.

Together with fostering gender balance in research teams and in decision-making, integrating the gender dimension in research and education is one of three key objectives for promoting gender equality in research and innovation in Europe [32]. Research shows that increasing the participation of women in research and innovation “*will not be successful without restructuring institutions and incorporating gender analysis into research*” [65, 66]. Grounded on the Stanford University project Gendered Innovations (http://genderedinnovations.stanford.edu/), the EC report Gendered Innovations: How Gender Analysis Contributes to Research [32] demonstrated how sex and gender analysis enhances the scientific quality, societal relevance and business value of research, and provided tools and guidance to do so.^4^ Since 2013, the EC has also supported the development of the European Gender Medicine Network, which provides a framework for the inclusion of sex and gender in health research. Furthermore, participation in Horizon 2020 funding requires that applicants describe how sex and/or gender analysis is considered in the content of the projects (https://ec.europa.eu/research/participants/docs/h2020-funding-guide/cross-cutting-issues/gender_en.htm).

Promoting the integration of sex and gender analysis in research and education represents a strategic opportunity to strengthen Athena SWAN in the given research-intensive study setting. There is a growing body of evidence on how the incorporation of sex and gender in research leads to better healthcare [67] or how the disregard of gender aspects [68–70] leads to suboptimal, sometimes harmful healthcare [52, 65, 71]. The world’s leading health research funder, the United States National Institutes of Health Research, made it mandatory in 2016 that all researchers account for sex as a biological variable [72]. Many other health research funders worldwide have also introduced policies that require that all grant applicants consider sex and gender variables in research design [73]. There is a pool of guidelines and toolkits to support the scientific community in taking into account sex and gender in research content, such as the IGAR Tool developed in the context of the European Research Area Network Gender-NET (http://igar-tool.gender-net.eu/en), which provides research funding organisations with screening of research proposals for sex and gender differences awareness, and the online training tools created by the Canadian Institute of Gender and Health (http://www.cihr-irsc.gc.ca/e/49347.html). Likewise, the European Association of Science Editors has introduced the Sex and Gender Equity in Research guidelines to maximise the generalisability and applicability of research findings to clinical practice [74]. The Sex and Gender Equity in Research guidelines help editors and researchers to ensure the adequate reporting of sex and gender information in study design, data analysis, results and interpretations of findings [75, 76].

Moreover, in a recent commentary on editorial policies, the *Lancet* proposed guidelines for medical journals, accounting for the use of sex and gender and reporting of sex, gender or both in study participants and the sex of animals and cells [77]. In 2015, the League of European Research Universities presented 20 recommendations on how to integrate sex and gender into the research process, research funding, curricula and clinical practice [78]. Including sex and gender analysis in the curricula of medical sciences courses helps students improve their study design, analysis and reporting skills [65], and gender-sensitive curricula, portraying gender in a non-stereotypical way, may make academic and research careers in medical sciences more attractive to all irrespective of gender [79].

## Discussion

### Athena SWAN as a complex social intervention

Despite a growing body of literature on the design, implementation and impact of gender equality interventions, there is a paucity of research on the complexity of gender equality interventions based on extensive empirical data [35]. To address this paucity of research, we embraced the complexity approach and analysed 16 departmental Athena SWAN Silver action plans in medical sciences at the University of Oxford.

Our analysis demonstrates that the Athena SWAN Silver action plans conform to the key considerations of complexity and thus can be usefully framed as complex social interventions embedded in a complex system.

Firstly, addressing a specific area of gender inequality is not enough in complex systems when designing gender equality interventions as a variety of interconnected factors are involved in the process [80]. The efficacy of gender equality interventions depends not only on the quality [12, 81] but also on the quantity of the measures implemented [82]. Athena SWAN provides a dynamic multifaceted systemic design and implementation process to address cultural and structural aspects of gender inequality in accordance with the needs, baseline conditions and emerging circumstances in the participating departments. Athena SWAN’s focus on the local dynamics in departments, a key characteristic of complex systems [12], is particularly important because departments rather than the central university make recruitment and promotion decisions, hold research funding and provide working environments. Overall, the 16 medical sciences departments implement 547 actions organised into five themes and 22 subthemes. Actions are tailored to the specific departmental contexts and vary greatly in design, target populations, areas of intervention and pace of implementation. Within departments, many actions are attuned to the context of different departmental divisions, institutes, centres, units and research groups. Given that most of the departments are embedded in the context of hospitals and clinical facilities, actions vary between different medical specialties and basic science areas. Moreover, departments are often distributed across several campuses and physical locations, adding another layer of complexity to action planning.

Secondly, the complexity approach embraces the notion of the non-linearity of interventions and the constantly emerging conditions [12, 35, 45]. The non-linear relationship between inputs, outcomes and impact of gender equality action plans depend on the interaction of a variety of variables dynamically related to contextual factors. As Greenhalgh and Papoutsi [40] state, instead of a linear, cause-and-effect causality, the complexity paradigm, characterised by emergent causality, where manifold interacting features and “*multiple uncertainties* [are] *involved*”, produces effects that cannot be ascribed to one particular influence. Therefore, the design of complex gender equality interventions cannot afford to underestimate the inconsistency and unpredictability of the implementation of the planned actions [12], in particular in self-organised contexts as the medical sciences departments in focus. Athena SWAN is not a stable arrangement but a dynamic system that is in a continuous interaction with the environmental conditions, addressing the constantly emerging conditions as self-organisation processes work against change and towards stabilising the system in the departments. Although initially Athena SWAN was set up to address barriers for women in academic and research roles, it has evolved to develop a broader focus on gender equality among all staff and students, taking into account local conditions and considerations of intersectionality such as sexuality, race, disability, age and religion. Strikingly, the target population of the Athena SWAN Silver actions analysed by gender are predominantly all genders indiscriminately (87.9%) and nearly a half of the actions (48.4%) target all staff, students, or professional and support staff. It is likely that multiple uncertain components in the analysed action plans would create an additional layer of complexity during their implementation. SATs continuously interact with the environmental conditions and address new emerging conditions. Namely, they monitor data and meet on a regular basis to map the outcomes of the action plans, evaluate feedback and redesign actions to address new conditions. Discussions and negotiations about different dimensions of change take place both internally within the departments and the university as well as externally within the wider system constituted by the higher education and research sector, the social, economic and political conditions, and the Athena SWAN community of practice.

Thirdly, the great number of variables involved in complex interventions, pointing out the persistent evolution of new variables in an adaptive system, highlight the numerous challenges for assessing impact in the studied departments [37]. In complex interventions, implementation, outcome and impact become even less predictable, manageable and responsive to linear logic [83]. Therefore, the contribution – rather than attribution and causality – of gender equality interventions to the outcome and impact is central in assessing Athena SWAN. Moreover, the complexity approach implies considering the increased probability of change as part of the desirable effect of complex interventions [12]. As a corollary, the expected impact of complex social interventions needs to be considered in terms of how they foster the conditions for change and increase the probability that change can occur in a particular complex setting as the one in the medical sciences division [35, 58]. The impact of Athena SWAN action plans is hence expected in terms of contribution to change, improved conditions to foster change, and the increased probability that change can occur in the departments in focus [35, 58].

The above discussed results provide examples of actions illustrating the wide range where impact can occur. In line with the complexity approach, we claim that design, implementation and impact of complex social interventions are best captured and assessed using a combination of quantitative and qualitative methods, case studies and illustrative examples. Only a small proportion of actions, such as those regarding recruitment, promotion and permanent contracts, aim to directly address the under-representation of women in certain positions. Therefore, the impact of only a small proportion of actions can be assessed using traditional quantitative indicators such as the number and proportion of women in certain positions. The majority of the implemented actions, especially those regarding organisation and culture, career development, and flexible working and career breaks, aim to improve conditions to foster change and increase the probability that change can occur. Moreover, a large number of actions regarding self-assessment and monitoring may also create emergent effects, such as the Hawthorne effect, whereby staff modify their behaviour in response to the awareness of being observed. Therefore, assessing the impact of the majority of actions would require a combination of quantitative and qualitative methods taking into account possible emergent effects.

### Implications for implementing and assessing the impact of Athena SWAN

While the assessment of the impact of the analysed Athena SWAN Silver actions is beyond the scope of this paper, the preceding discussion on Athena SWAN as a complex social intervention presents us with a range of practical implications for the implementation and impact assessment^5^ of the scheme.

As regards implementation, by using a complexity approach, agents avoid a reductionist stance and gain new insights into addressing and managing the dynamic process of Athena SWAN, which requires investing into recruiting and developing highly qualified local implementation professionals with the capability and capacity to handle and dynamically respond to new emergent conditions and changes in the environment. Thus, implementation professionals would need to be able to move away from a model that accounts for how the parts contribute to the whole towards a model that tries to understand how each part interacts with all the other parts to emerge as a new entity by looking at the multiple interrelated elements [84]. This would allow a comprehensive understanding of the whole in the localness of each implementing department, i.e. looking into its physical, social, economic and political elements. This approach would help identify and address the emerging conditions because the departments constantly adapt to change and evolve towards self-organisation and order [36]. Accordingly, accounting for the influence of the context and the local dynamics [85, 86], and the opening space for new possibilities, the implementation professionals would need to frequently develop new implementation techniques and methods [87] and employ actions suitable for the local emergent conditions. Emergent conditions are closely related to how actors in the departments interpret actions and situations and demonstrate the lack of complete control over the outcomes [88]. Furthermore, it is likely that implementation professionals would become aware of the fact that, what seems to be a dominant cause of inequality in a certain department at one point in time, might shift later due to the constant interplay of a multitude of contextual factors [35]. Therefore, as Greenhalgh and Papoutsi [40] state “*we need to develop capability and capacity to handle the unknown, the uncertain, the unpredictable and the emergent*” [89].

Recruiting and developing highly qualified local implementation professionals with the capability and capacity to handle and dynamically respond to new emergent conditions is particularly important for addressing the unintended consequences and perverse incentives that have emerged during the implementation of Athena SWAN in United Kingdom higher education institutions. For example, qualitative research found that women were disproportionally involved in Athena SWAN SATs and bore the administrative burden of preparing Athena SWAN applications and action plans [14, 23, 28]. Due to the pressures to achieve gender balance on committees and panels as required for Athena SWAN awards, the few senior women were overstretched with administrative duties and participation on various committees and panels [14], possibly to the detriment of their research and teaching. Another important unintended consequence is the emergence of “*competing inequalities*”, with the Athena SWAN Charter inadvertently taking prevalence over the Race Equality Charter [26]. Research shows that, despite the importance of addressing intersectionality in Athena SWAN applications, there is little imperative in United Kingdom higher education institutions to address racism and classism [26]. While white middle class women are considered to be the main beneficiaries of the Athena SWAN Charter [27], a racially diverse profile at some institutions appears to be achieved thanks to highly privileged overseas academics rather than home working class black and minority ethnic academics [20].

As regards impact assessment, there are a number of implications to consider in relation to Athena SWAN. Firstly, widening the areas where impact can be recognised; this requires going beyond reductionist approaches, what traditionally is perceived as impact – and often measured quantitatively – to also include qualitative methodologies and parameters. Being a complex system, Athena SWAN cannot be fully assessed and analysed using traditional techniques and linear causal approaches [90].

Secondly, considering the ability of interventions to produce impact within the targeted areas in the departments in focus. According to the complexity perspective, every Athena SWAN intervention is locked into a social, institutional, socioeconomic and political system, and insight into how these facilitate or hinder the input–impact chain is necessary to understand impact [88]. Thus, it is important to anticipate the ability of the design and implementation of Athena SWAN interventions in the departments to foster the enabling “*conditions for change*” linking the design, implementation and effect of interventions to adequate conditions to produce impact [58]. This means that each medical science department and its ability to achieve change in accordance with the Athena SWAN scheme is very different from other departments and has to be assessed in its own local environment.

Thirdly, since linear effects are difficult to establish in such a complex scheme as Athena SWAN, taking a probabilistic approach identifying potential impacts on the creation of the right conditions for change in a medium- and long-term perspective is central to impact assessment. This probabilistic stance makes impact assessment of complex interventions “*less deterministic and more substantive*” [12]. Probability can then be assessed “*through a set of indicators pointing to the activation of internal change processes (e.g. the successful involvement of internal and external actors, the modification of relevant rules, and the creation of internal groups of actors aimed at pursuing change), as internal processes are likely to produce additional impacts with time*” [35]. As we address non-linear processes where impact is not predictable, small changes in the studied departments can have large impact while large changes can lead to limited success [90]. There is thus a disproportionality between the Athena SWAN intervention and its effect. This implies that policy-makers cannot expect to measure the direct links between the intervention and its impact but, as mentioned above, have to account for the contribution of the scheme to achieving impact. In practice, this would require a greater emphasis on process indicators versus outcome measures.

### Strengths and limitations of the study

To the best of our knowledge, this is the first study to empirically show that Athena SWAN is a complex social intervention and to discuss its implications for policy and practice. The study is based on the most extensive dataset of Athena SWAN interventions in medical sciences, but it is limited to a single site. Extending data collection and analysis to multiple sites is likely to capture a greater range of interventions and contextual factors. Another unique contribution of this study lays in comparing Athena SWAN Silver interventions with the EFFORTI typology of gender equality interventions in research and innovation in the wider European Research Area. Having a comparative European perspective can help generate insights on the strengths, limitations and opportunities for further development of Athena SWAN. Given the current policy interest in introducing a gender equality award scheme similar to Athena SWAN in the wider European Research Area, our comparative analysis has the potential to inform policy and practice wide across Europe.

## Conclusions

To activate effective gender equality structural and cultural change, it is necessary to acknowledge and operationalise the notion of complexity as a frame of reference. Athena SWAN is the single most comprehensive and inclusive gender equality scheme in Europe. It can be further strengthened by promoting the integration of sex and gender analysis in research and education. Gender equality policies in the wider European Research Area can benefit from exploring Athena SWAN’s contextually embedded systemic approach to action planning and inclusive focus on all genders and categories of staff and students.

## Supplementary information

**Additional file 1.** Overview of the EFFORTI typology of gender equality interventions in research and innovation (source Kalpazidou Schmidt et al. [59]).

**Additional file 2.** Departmental codes.

**Additional file 3.** Departmental Athena SWAN action plans.

## Data Availability

The departmental Silver award applications and action plans analysed during the current study are publicly available on the departmental websites and also provided in Additional file 3.

## Abbreviations

EC: European Commission
EFFORTI: Evaluation Framework for Promoting Gender Equality in Research and Innovation
NHS: National Health Service
NIHR: National Institute for Health Research
PDR: personal development review
SAT: self-assessment team

## Footnotes

1 A "Made-in-Canada" adaptation of the Athena SWAN scheme, reflecting post-secondary institutions, called ‘Dimensions’ is a tri-agency initiative led by the Natural Sciences and Engineering Research Council. It aims at advancing research excellence and innovation with a focus on equity, diversity and inclusion. Participation in the programme is voluntary; 17 institutions are being involved in the first pilot cohort. The participant institutions are given 2 years to prepare their self-assessment report and develop their applications (https://www.nserc-crsng.gc.ca/NSERC-CRSNG/EDI-EDI/Athena-SWAN_eng.asp).

2 Responsible Research and Innovation (RRI) is promoted through actions on thematic elements (public engagement, gender, open access, ethics, science education) as well as integrated actions that address institutional change to foster the uptake of the RRI approach by stakeholders and European organisations. For a more detailed description of the RRI concept see ‘European Commission. Responsible Research and Innovation. Europe’s ability to respond to societal challenges. European Union. 2012, Brussels. ( https://ec.europa.eu/research/swafs/pdf/pub_rri/KI0214595ENC.pdf).’

3 An interesting United Kingdom study containing 50 potential interventions representing good practice or positive action and addressing cultural, organisational and individual barriers to gender equality, ranked by participants according to their perception of priority, is presented in a typology developed by Bryant et al. [24].

4 Practical methods of sex and gender analysis for researchers have been developed and checklists have been provided by experts. The Gendered Innovations project has, for instance, developed practical examples of how sex and gender analysis leads to gendered innovations in science, health and medicine, engineering and the environment (see http://genderedinnovations.stanford.edu/).

5 The strategies are developed from Kalpazidou Schmidt and Cacace [35].
