# Supplementary material for: Understanding the Athena SWAN award scheme for gender equality as a complex social intervention in a complex system: analysis of Silver award action plans in a comparative European perspective
Source: Health Res Policy Syst. 2020 Feb 14;18:19. doi: 10.1186/s12961-020-0527-x (PMC7023775; doi:10.1186/s12961-020-0527-x)
Supplement: Supplementary file 1 — Additional file 1. Overview of the EFFORTI typology of gender equality interventions in research and innovation (source Kalpazidou Schmidt et al. [59]). [file 12961_2020_527_MOESM1_ESM.docx]

**Additional file 1.** Overview of the EFFORTI typology of gender equality interventions in research and innovation (source Kalpazidou Schmidt et al. 2018)

| **Type of intervention** | **Intervention format** |
| --- | --- |
| 1. **Policies** | 1. Mainstreaming actions 2. Gender equality/action plan 3. Gender budgeting |
| 1. **Non-discrimination** | 1. Gender-sensitive practices for the attribution of tasks 2. Gender-sensitive study and working conditions (e.g. alternative study plans for pregnancy during laboratory work period) 3. Gender-sensitive Human Resources management 4. Guidelines regarding gender specifics |
| 1. **Composition & Integration** | 1. Definition of targets regarding gender balance in decision-making positions 2. Definition of targets regarding gender balance in research groups 3. Institution of quotas |
| 1. **Advancement** | 1. Mentoring programmes 2. Gender-sensitive practices for assessment 3. Introduction of chairs and positions reserved to women 4. Support to career development (counselling) 5. Empowerment schemes |
| 1. **Recruitment** | 1. 16. Campaigns for inspiring women for Mathematics, information technology, natural sciences and technology (MINT) subjects |
| 1. **Monitoring** | 1. Monitoring appointments, promotions, or attributions of tasks |
| 1. **Deconstructing Excellence** | 1. Revision of internal policies regarding promotions 2. Revision of internal policies regarding staff appointments |
| 1. **Gender Awareness & Bias** | 1. Training courses (different targets) |
| 1. **Leadership Accountability** | 1. Implementation of gender-sensitive leadership and personnel development |
| 1. **Funding** | 1. Targeting funding practices to improve women’s access to research funding 2. (Targeted) funding to improve the integration of gender dimension in research 3. Targeted funding practices to encourage research organisations to promote gender equality measures 4. Special funding for women researchers |
| 1. **Research** | 1. Gendered user involvement 2. Inclusion and monitoring the integration of the gender dimension and impact |
| 1. **Knowledge** | 1. Dissemination of information material 2. Revision of teaching curricula and texts 3. Introduction of single-sex degree and specialisation courses 4. Provision of gender and women studies or modules 5. Integrating the gender dimension in tertiary education |
| 1. **Visibility** | 1. Networking 2. Activities to make women (and their research) visible (e.g. introduction of awards reserved for women) 3. Role models |
| 1. **Care & Family Life** | 1. Support in period of absence for family needs 2. Schemes for women returners 3. Care services and facilities (for children, the elderly, and others) 4. Support to mobility, including spouse relocation schemes |
| 1. **Work-Life Balance** | 1. Introduction of flexible working hours |
