# Supplementary material for: Understanding the Athena SWAN award scheme for gender equality as a complex social intervention in a complex system: analysis of Silver award action plans in a comparative European perspective
Source: Health Res Policy Syst. 2020 Feb 14;18:19. doi: 10.1186/s12961-020-0527-x (PMC7023775; doi:10.1186/s12961-020-0527-x)
Supplement: Supplementary file 3 — Additional file 3. Departmental Athena SWAN action plans. [file 12961_2020_527_MOESM3_ESM.docx]

**Additional file 3.** Departmental Athena SWAN action plans.

<https://oxfile.ox.ac.uk/oxfile/work/extBox?id=8184950BF79422502C>
